# A survey identifying nutritional needs in a contemporary adult cystic fibrosis cohort

**DOI:** 10.1186/s40795-018-0266-3

**Published:** 2019-01-07

**Authors:** Siddhartha G. Kapnadak, Kathleen J. Ramos, Andrea M. Lopriore, Christopher H. Goss, Moira L. Aitken

**Affiliations:** 1 Department of Medicine; Division of Pulmonary, Critical Care, and Sleep Medicine, University of Washington Medical Center, 1959 NE Pacific, Campus Box 356522, Seattle, WA 98195-6522 USA; 2 Department of Nutrition, University of Washington Medical Center, 1959 NE Pacific, Campus Box 356522, Seattle, WA 98195-6522 USA; 3 Department of Pediatrics; Division of Pulmonary and Sleep Medicine, University of Washington Medical Center, 1959 NE Pacific, Campus Box 356522, Seattle, WA 98195-6522 USA

**Keywords:** Cystic fibrosis, Complications, Malnutrition, Body mass index, Body composition, Nutrition assessment, Nutrition therapy, Obesity

## Abstract

**Background:**

Cystic fibrosis (CF) is a disease in which nutritional barriers are diverse and common, with malnutrition greatly influencing pulmonary trajectory and overall outcomes. Despite this, the most effective methods to optimize CF nutrition are unknown, and literature describing patients’ perspectives on their specific nutritional needs is lacking, particularly in the modern era of CF care. This study aimed to identify the most important nutritional needs and desired health-improvement resources in a contemporary adult CF cohort.

**Methods:**

A 14-question investigator-designed survey addressing nutrition concerns, preferred health-improvement resources, and dietary/exercise routines was administered to CF adults. Clinical characteristics and survey responses are presented with descriptive statistics, and responses compared by body mass index (BMI) category (< 18.5 kg/m^2^; 18.5–24.99 kg/m^2^; 25–29.99 kg/m^2^; ≥30 kg/m^2^), gender, and socioeconomic status using Chi square or Fisher’s Exact testing.

**Results:**

Of 66 total patients, nine (13.6%) were underweight (BMI < 18.5 kg/m^2^), while 19 (28.8%) were overweight or obese (BMI ≥ 25 kg/m^2^). In the overall cohort, the most common primary concern was preventing weight loss [in 20/66 patients (30.3%)], but there were significant differences by BMI (*p* < 0.001), with the most common concern in the overweight subgroup being preventing weight gain. Fifteen (46.9%) men (BMI mean 20.7, range 16.4–29.2 kg/m^2^) listed preventing weight loss as the primary concern, compared to only 5 (14.7%) women (BMI mean 18.4, range 16.2–19.9 kg/m^2^), representing a trend toward a difference in primary concerns by gender (*p* = 0.066).

The most commonly desired health-improvement resource was online CF nutrition and fitness information, found in 26 patients (39.4%) in the overall cohort, without significant differences by BMI (*p* = 0.814) or gender (*p* = 0.199). Financial assistance was the preferred resource in 17 (26.2%), without differences by socioeconomic status (*p* = 0.367).

**Conclusions:**

We identified a wide variety of nutritional needs in CF adults, including a high prevalence of overweight status, many patients desiring weight loss, and many seeking financial resources. Our findings support the individualization of modern-day CF nutrition programs and development of online resources, in an effort to address the heterogeneous barriers that exist in the contemporary CF population and improve outcomes in patients with the disease.

**Electronic supplementary material:**

The online version of this article (10.1186/s40795-018-0266-3) contains supplementary material, which is available to authorized users.

## Background

Cystic fibrosis (CF) is a multisystem disease caused by mutations in the cystic fibrosis transmembrane regulator (CFTR) gene, and represents the most common life-shortening autosomal recessive disease in Caucasians [1]. Although 70% of mortality in CF is due to progressive lung disease, CFTR dysfunction can result in a high incidence of pancreatic exocrine insufficiency, gastrointestinal symptoms, CF-related diabetes mellitus (CFRD), and other clinically important non-pulmonary sequelae. With improved management of CF lung disease and survival, extra-pulmonary conditions have become an increasingly important cause of morbidity for patients with CF [2, 3].

Of the CF-associated extra-pulmonary manifestations, it can be argued that malnutrition is among the most critical. Malnutrition in CF, standardly defined as a low body mass index (BMI), has been found to be multifactorial, common, and independently associated with mortality, decreased quality of life and lung function, and poor outcomes after lung transplant [4–11].

Recognizing the significance of nutrition, the CF Foundation (CFF) has developed BMI-based nutritional goals for all patients: BMI ≥ 50th percentile in children, ≥ 22 kg/m2 in adult women; and ≥ 23 kg/m2 in adult men. Since the 1980’s, significant progress has been made in optimizing nutrition in the CF population, but many individuals fall short of these goals [3, 5, 12, 13]. Moreover, although the CFF hasn’t defined upper limits to BMI goals, recent data have shown that overweight status is also a concern in the contemporary CF population, particularly in patients with milder mutations [3, 14, 15]. Perhaps related to aggressive caloric supplementation, suboptimal food choices, lack of exercise, and medications, this has added an additional layer of complexity to modern CF nutritional management.

Despite the importance in CF, there are limited data on how best to optimize nutrition and fitness, particularly in the growing adult demographic which now makes up more than 50% of the CF population. Although multidisciplinary programs have been suggested [5, 16], it is not clear which interventions are most effective, and it has been demonstrated that dietitians vary in their clinical approaches [17–19]. Furthermore, it is clear that nutritional barriers vary significantly based on the individual, with patients facing a wide array of problems from medical issues to specific knowledge gaps and socioeconomic barriers [20, 21]. In this sense, identifying concerns on an individualized basis, and implementing patient-centered nutrition programs may be the most effective way to optimize adherence, perceptions of body image, and overall nutrition [17, 22]. However, to this point there is minimal literature describing CF patients’ perspectives regarding their nutritional and fitness needs, nor is there information outlining nutrition programs designed based on patient preferences [20, 23, 24].

The objective of this study was to identify the most important nutritional concerns and desired health-improvement resources in a contemporary cohort of CF adults. Secondarily, we aimed to determine whether these identified needs differed based on specific patient characteristics including body mass index, gender, and socioeconomic status.

## Methods

### Setting

This study began as a prospective quality improvement project in our adult CF clinic, which serves patients ≥18 years of age in the Northwestern United States. All outpatients seen from March 2016–August 2016 were eligible and were given the choice to participate. For patients choosing to participate, because it began as a quality improvement project, the University’s Institutional Review Board did not require formal consent for prospective data collection; retrospective data review for the study was later approved without patient consent.

Clinical care in our center was per CFF guidelines, with patients evaluated every 3 months by a CF physician and at least annually by a CF-trained registered dietitian (RD). All visits included a weight measurement (BMI calculated in kg/m^2^), history and examination, and medication review. Diabetes screening and assessment of vitamin levels was performed at least annually.

The following data were prospectively collected at the time of survey response: age, gender, BMI, forced expiratory volume in 1 s (FEV_1_), and diabetes status. CFTR genotypes were obtained from the local CFF patient registry portal. As in many other studies, receipt of United States Medicaid insurance (which provides funding for those with limited income) was used as a proxy for low socioeconomic status [25].

### Survey

A 14-question survey was created to identify the most important nutritional concerns and desired health-improvement resources in our cohort (Additional file 1). The survey was designed by three CF physicians and our center’s CF dietitian. All four providers personally completed the survey and edited it for completeness, clarity, and simplicity prior to implementation.

The survey was offered to all patients seen by the RD, with the same RD consistently available for questions encountered during completion. Primary nutrition concerns were assessed by rankings of the following options: 1) Preventing weight loss (or promoting weight gain); 2) Nutrition and food choice education; 3) Blood sugar management; 4) Preventing weight gain (or promoting weight loss); 5) Digestive health/pancreatic enzymes. Patients were also offered choices of health-improvement resources including: 1) Online access to CF nutrition and fitness information; 2) A CF cookbook based on unprocessed foods; 3) Financial assistance for food and supplements; 4) Access to local food resources (e.g., food banks, meal programs).

Time spent on food preparation, exercise frequency/duration, and questions surrounding medication use were evaluated with multiple choice questions. Patients were also asked to rate the utility of dietitian visits in the context of the patient-centered nutrition program, using a Likert scale from 1 (very helpful) to 5 (not helpful).

### Statistics

Patient characteristics and survey responses were represented with descriptive statistics. Differences were assessed with student’s t-test for continuous variables, allowing for unequal variances, and with Chi square or Fisher’s Exact testing for categorical variables for patients with different CFTR mutation classes (Class I-III versus Class IV-V mutations). For survey responses to the main questions of interest (primary nutrition concern and preferred health-improvement resource), differences in outcome were assessed by BMI category (< 18.5 kg/m^2^; 18.5–24.99 kg/m^2^; 25–29.99 kg/m^2^; ≥30 kg/m^2^), gender, and Medicaid insurance status using Chi square or Fisher’s Exact testing.

## Results

### Demographics

A total of 66 patients (34 women, 32 men) were offered the survey during the quality improvement project and all completed it (100% survey response rate). Fifty-four (81.8%) were classified as having severe CF genotypes (CFTR Class I-III) (Table 1). The most common mutation was F508del, with 27 patients (40.9%) homozygous, and 36 (54.5%) heterozygous. Mean age was 32.3 years, and mean FEV_1_ was 59% predicted.

**Table 1:** Patient Characteristics by Cystic Fibrosis Transmembrane Regulator Mutation Status

	All Eligible Patients *N* = 66		CFTR Mutation Class I-III *N* = 54		CFTR Mutation Class IV-V *N* = 12		
Variable	Observed	N	Observed	N	Observed	N	*p* Value
Age – mean (SD), years	32.3 (11.4)	66	30.4 (8.8)	54	40.8 (17.2)	12	0.0637
Male gender	32 (48.5)	66	27 (50.0)	54	5 (41.7)	12	0.601
Medicaid Insurance	23 (35.4)	65	17 (32.1)	53	6 (50.0)	12	0.241
CFTR mutation status		66					
Homozygous Class I-III	54 (81.8)						
Heterozygous Class I-III	12 (18.2)						
Homozygous Class IV-V	0 (0)						
FEV_1_ - % predicted (SD)	59.3 (23.3)	66	57.3 (22.7)	54	68.3 (24.9)	12	0.178
Weight – mean (SD), kg	66.1 (14.6)	66	63.9 (11.9)	54	76.0 (21.2)	12	0.079
BMI – mean (SD), kg/m^2^	23.4 (4.3)	66	22.8 (3.5)	54	26.2 (6.4)	12	0.099
BMI < 18.5	9 (13.6)	66	7 (13.0)	54	2 (16.7)	12	0.663^b^
BMI 18.5–24.99	38 (57.6)	66	34 (63.0)	54	4 (33.3)	12	0.104^b^
BMI 25–29.99	13 (19.7)	66	11 (20.4)	54	2 (16.7)	12	1.000^b^
BMI ≥ 30	6 (9.1)	66	2 (3.7)	54	4 (33.3)	12	**0.008** ^b^
BMI meeting CFF goal^a^	38 (57.6)	66	29 (53.7)	54	9 (75.0)	12	0.177^b^
CF-related diabetes on insulin	15 (23.1)	65	14 (26.4)	53	1 (8.3)	12	0.267^b^
CF-related PI on enzymes	58 (89.2)	65	52 (98.1)	53	6 (50.0)	12	**< 0.001** ^b^
Prednisone use (at time of survey)	5 (7.7)	65	5 (9.4)	53	0 (0)	12	0.575^b^
PEG tube in place	2 (3.1)	65	1 (1.9)	53	1 (8.3)	12	0.338^b^
Nutritional supplement use	32 (49.2)	65	26 (49.1)	53	6 (50.0)	12	0.953

Data are presented as No. (%) unless indicated otherwise. Certain background clinical data were not recorded and were thus unavailable for one individual

^a^CFF goal for weight is BMI ≥22 kg/m^2^ for females, and ≥ 23 kg/m^2^ for males

^b^= Fisher’s exact test

*CF* cystic fibrosis, *SD* standard deviation, *CFTR* cystic fibrosis transmembrane conductance regulator gene, *FEV1* forced expiratory volume in one second, *BMI* body mass index, *CFF* Cystic Fibrosis Foundation, *PI* pancreatic insufficiency, *PEG* percutaneous endoscopic gastrostomy

Pancreatic insufficiency was more common in those with severe genotypes, with 98.1% of patients with class I-III mutations taking pancreatic enzyme replacement therapy, compared to 50.0% of patients with class IV-V mutations (*p* < 0.001). Mean BMI in the overall cohort was 23.4 kg/m^2^, without statistical difference by CFTR genotype (*p* = 0.099).

Thirty-eight patients (57.6%) had BMIs meeting CFF goals (≥ 22 kg/m^2^ for women, ≥ 23 kg/m^2^ for men). Only nine patients (13.6%) were underweight (BMI < 18.5 kg/m^2^). Conversely, overweight status was relatively common, with 19 patients (28.8%) having BMI ≥ 25 kg/m^2^. Obesity (BMI ≥ 30 kg/m^2^) was more common in those with mild mutations [4 of 5 patients (33%) with mild mutations versus 2 of 54 (3.7%) patients with severe mutations, *p* = 0.008].

### Overall survey data

Survey completion time was < 10 min for all 66 patients. Participants rated the patient-centered RD program highly, with mean score 2.1 out of 5, where 1 indicated “very helpful”.

Time spent on food preparation varied widely, with 19 patients (29.2%) spending greater than 45 min daily, 19 (29.2%) 31–45 min daily, 24 (36.9%) 11–30 min daily, and 3 (4.6%) 0–10 min daily.

Patients reported exercising a mean of 3.6 days/week, with a majority reporting duration of > 30 min per session. Specific exercise patterns varied, with 11 (19.6%) patients reporting mean exercise durations > 60 min daily, and 6 (10.7%) reporting durations 0–15 min daily. Walking, running, and strength training were the most popular forms of exercise.

### Primary nutrition concerns and choice of health-improvement resources

**Fig. 1:**
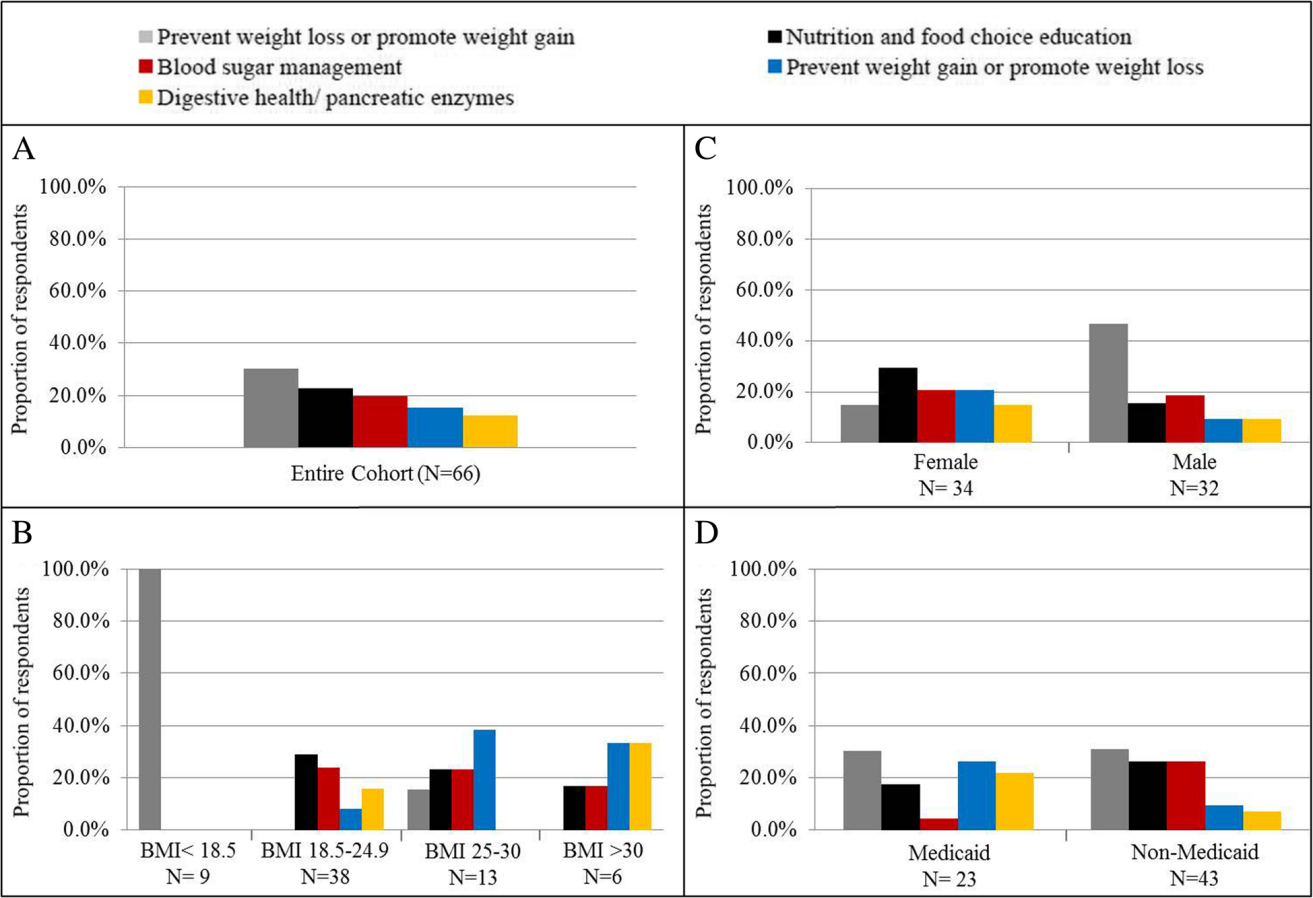
Primary nutrition concerns. **a** In the entire cohort; **b**) By BMI (in kg/ m^2^); **c**) By gender; **d**) By insurance status, (BMI = Body mass index)

In the entire cohort, the most common primary nutrition concern was preventing weight loss, found in 20 (30.3%) of respondents (Fig. 1a). Nutrition education was also a common response, answered in 15 (22.7%) patients in the cohort.

**Fig. 2:**
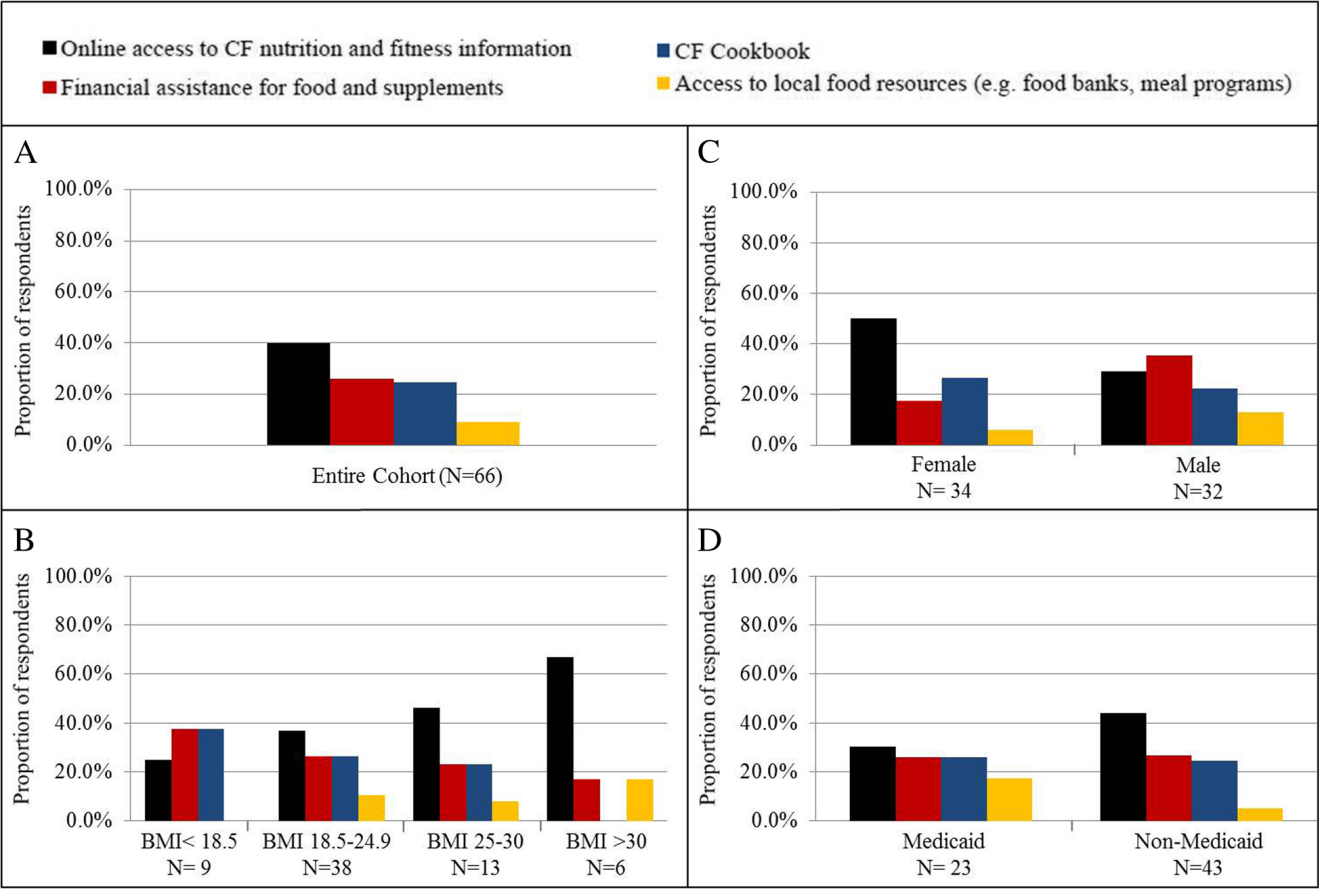
Preferred health-improvement resources. **a** In entire cohort; **b**) By BMI (in kg/m^2^); **c**) By gender; **d**) By insurance status, (BMI = Body mass index, CF = Cystic fibrosis)

The most commonly sought out health-improvement resource in the overall cohort was online access to CF nutrition and fitness information, with 26 patients (39.4%) listing this as their preferred choice of the offered programs (Fig. 2a). Seventeen patients (26.2%) listed financial assistance for food and supplements as their preferred choice.

### Differences by body mass index

Differences in primary nutrition concern by BMI category are displayed in Fig. 1b. All patients with BMI < 18.5 kg/m2 (*n* = 9) reported preventing weight loss as their primary concern. In contrast, in patients with BMI 25–29.99 (*n* = 13) and ≥ 30 kg/m2 (*n* = 6), preventing weight gain was the most common concern, found in 38.5 and 33.3% of patients, respectively (*p* < 0.001 for comparisons by BMI category, Fisher’s Exact testing).

Differences in preferred health-improvement resources are displayed in Fig. 2b. Financial assistance was a more common response in underweight patients than the overall cohort, found in 37.5% of respondents with a BMI < 18.5 kg/m^2^. In patients with BMI ≥ 30 kg/m^2^, 66.7% listed online access to CF nutrition and fitness information as their top program choice. Despite these qualitative differences compared to the overall cohort, there were no significant differences in choice of health improvement resources by BMI status (*p* = 0.814).

### Differences by gender

Mean BMI was 23.1 kg/m^2^ in women and 23.7 kg/m^2^ in men. The most common primary nutrition concern among women was nutrition education, found in 29.4% of female respondents (Fig. 1c). Among men, preventing weight loss was the most common primary nutrition concern, and was a much more common response in men (46.9% of respondents) compared to women (14.7% of respondents). Conversely, preventing weight gain was a more common response among women, listed as the primary nutrition concern in 20.6% of women compared to 9.4% of men. This represented a trend toward, but not a statistical difference in primary nutrition concerns by gender (*p* = 0.066).

The preferred health-improvement resources also varied qualitatively by gender, with 50.0% of women listing online access to CF nutrition and fitness information as their preferred program, compared to 29.0% of men (Fig. 2c). The most commonly sought out resource in men was financial assistance for food and supplements, found in 35.5% of male respondents. Despite the qualitative differences, there was no statistical difference in preferred health-improvement resources by gender (*p* = 0.199).

### Differences by socioeconomic status

Of 66 patients in the entire cohort, 23 (34.8%) were on Medicaid. Mean BMI was 23.8 kg/m^2^ in the Medicaid subgroup and 23.1 kg/m^2^ in the Non-Medicaid subgroup *(p* = 0.582). Underweight status was more common in the Medicaid subgroup, with 21.7% of Medicaid patients having BMI < 18.5 kg/m^2^, compared to only 9.5% of patients in the non-Medicaid subgroup. Additionally, obesity was more common in the Medicaid subgroup, with 17.4% of Medicaid patients having a BMI > 30 kg/m^2^, compared to only 4.8% of patients in the non-Medicaid subgroup. Despite these trends, comparisons did not meet statistical significance (*p* = 0.260 and *p* = 0.174, respectively).

Preventing weight loss was the most common nutrition concern in both the Medicaid and non-Medicaid subgroups, but the percentage of patients who were most concerned about preventing weight gain was significantly higher in the Medicaid subgroup, found in 26.1% of Medicaid compared to 9.5% of non-Medicaid respondents (*p* = 0.048) (Fig. 1d).

In both the Medicaid and non-Medicaid subgroups, the most commonly chosen health-improvement resource was online access to CF nutrition and fitness information. Surprisingly, financial assistance for food and supplements was the preferred resource for a similar percentage of non-Medicaid (26.8%) and Medicaid (26.1%) patients, indicating no difference in health-improvement resource preferences by insurance status (*p* = 0.367).

## Discussion

Improving nutrition is a critical component of CF care, with malnutrition having clear associations with poor outcomes in patients with the disease [4, 5, 10, 11]. Classically, this has been targeted through a combination of increasing caloric intake and supplements, optimizing pancreatic enzyme replacement, and pulmonary/medical management, with general aim of maintaining BMI above CFF goals [5, 14]. Although multidisciplinary nutrition programs are suggested [5], to this point there is limited knowledge regarding the optimal methods or programs specifically desired by patients. In this study, we describe a patient-centered model that identified specific nutritional concerns and desired programs in a contemporary population of CF adults. The program was popular among participants and identified heterogeneous concerns, nutritional barriers, and desired interventions based on patient-specific factors in our cohort.

There are many barriers to achieving optimal nutrition in patients with CF [5, 9]. In addition to pancreatic exocrine insufficiency, present in at least 85% [3], CF patients have been shown to have markedly increased basal energy expenditure, a problem related to chronic pulmonary infection which can worsen in more advanced stages of lung disease. Additional medical barriers include dyspnea and mechanical effects of lung hyperinflation, gastrointestinal symptoms, and CFRD. Together with weight loss, lean body mass can also be disproportionately impacted by suboptimal dietary choices and lack of exercise, the latter of which may worsen in those with advanced lung disease [26–29]. Furthermore, psychosocial obstacles may be more prevalent in CF, including depression, low socioeconomic status, medical knowledge gaps, and issues with adherence to medical/nutritional programs [3, 21, 30–34].

Given the complexity of these barriers, it is natural that poor nutritional status in CF would be a multifaceted problem. Our findings are consistent with this notion. Importantly, in our cohort of CF adults, we found a high prevalence of overweight status. Although more than half met traditional CFF BMI goals, 28.8% were overweight, and 9.1% obese. This finding is consistent with previously demonstrated longitudinal trends in CF [14], with our percentages even exceeding prior estimates of the prevalence of overweight status [3, 15]. Furthermore, in our study, a significant portion of patients actually desired weight loss, with about 15% of the cohort listing this as their primary nutritional concern, a percentage that approached 40% in patients with a BMI > 25 kg/m^2^. The problem of CF-associated obesity, on one hand, may reflect improvements in pulmonary management and a general ageing of the CF population, both of which can be considered strong accomplishments in the disease’s history. On the other hand, although general CF nutritional trends have improved with average BMI in adults now above goal, the prevalence of obesity in our study supports the concept that the modern era of CF nutrition has evolved considerably. Recognition of the heterogeneity of nutritional barriers may become even more important as the CF population continues to age and cardiovascular risks increase [35, 36], where personalizing nutrition programs may become integral to improving CF outcomes.

The potential benefits of a patient-centered nutrition program approach are broad. Firstly, it has been demonstrated that CF patients respond well to assuming more control of their treatment plans, including recent data showing effectiveness of online and smartphone-based health-management programs [37, 38]. Secondly, there are studies showing that body image is an important component of well-being for CF children, adolescents, and adults and that improving body satisfaction may increase both adherence and quality of life [22, 39–42]. Body satisfaction is naturally influenced by subjective medical and psychosocial factors, with optimal satisfaction likely best attained by first identifying the specific barriers at hand [22, 43]. In our cohort we did find an array of nutritional and exercise habits, goals, and desired resources. As with previous reports, we identified gender differences in weight goals, with CF men more often concerned about preventing weight loss, and women more often concerned about preventing weight gain [41, 43]. Online access to CF nutrition and fitness resources was consistently the most sought-after health improvement resource, but many patients preferred other programs including direct education from CF dietitians, recipes via a CF cookbook, or financial assistance. The variety of needs presented in this study support the assertion that individualizing CF nutrition programs may be a valuable means of improving overall nutrition outcomes.

An important consideration in the patient-centered model presented here are financial and psychosocial barriers. Access to insurance coverage and financial limitations pose a challenge to many CF patients, with CFF registry data demonstrating that nearly half of individuals receive some portion of coverage through federal or state-funded programs, and low socioeconomic status having been associated with a variety of adverse outcomes [3, 21, 31, 44]. In our Medicaid cohort, although mean BMI was 23.8 kg/m^2^, it is noteworthy that nearly 60% fell outside of the “optimal” BMI range of 18.5–25 kg/m^2^, including roughly 35% overweight or obese, and 22% underweight. More than 1/3 of our cohort was on Medicaid, further suggesting that this may be a CF subset of particular importance when trying to optimize overall nutritional outcomes. In our study, financial assistance and local food resources were commonly desired health-improvement resources even among non-Medicaid patients, lending additional credence to the importance of addressing financial barriers in every patient. Especially considering the expenses and time constraints related to CF care, patient-centered nutrition programs would likely benefit from early assessment of financial concerns, along with education and other psychosocial barriers as they pertain to nutrition and fitness.

There were several limitations of this study, the first of which was a small sample size. The lack of statistically significant differences between subgroups (gender, BMI, socioeconomic status) may have been due to the study being underpowered. Larger studies on the topic are needed, but this exploratory analysis is the first to identify specific, individual nutrition concerns in a modern-day CF adult cohort, and thus represents a foundation on which further patient-centered nutrition programs may be developed. Second, this study used a convenience sample of adults attending our CF clinic with, on average, moderate lung disease. Although identifying nutritional needs may be a valuable tool in all CF cohorts, our specific findings are thus less generalizable to CF children, as well as in adults with very severe lung disease or post-transplant, where specific findings may differ. Likewise, in regards to socioeconomic comparisons, our study necessarily examined CF patients within the United States healthcare system, and while socioeconomic obstacles exist globally, the specific findings may vary in other systems. Finally, this study used an investigator-designed survey tool that has not been validated. Given that (to our knowledge) no similar validated tools exist, we consider it a first step in determining CF patient-specific nutritional needs, but since it was not an interventional study, our data cannot be used to determine the effectiveness of the suggested interventions. Future studies are needed to apply patient-specific interventions in CF using similar tools, while also examining nutritional and overall outcomes.

## Conclusions

In summary, in this study we describe nutritional demographics and concerns in a contemporary cohort of CF adults, finding a relatively high prevalence of overweight status, and recognizing a wide variety of barriers to optimizing nutrition. We describe a patient-centered model that allowed identification of specific concerns, goals, and desired health-improvement resources. The problem of overweight status in CF adults should be appreciated, and future studies are needed to implement, refine, and assess the efficacy of patient-centered programs, with a goal of improving nutritional and overall outcomes in patients with CF.

## Additional file

Additional file 1: Survey of nutritional needs. (DOCX 24 kb)

## Abbreviations

BM: Body mass index
CF: Cystic fibrosis
CFF: Cystic fibrosis foundation
CFRD: Cystic fibrosis related diabetes
CFTR: Cystic fibrosis transmembrane regulator
FEV_1_: Forced expiratory volume in one second
PEG: Percutaneous gastrostomy
PI: Pancreatic insufficiency
RD: Registered dietitian
SD: Standard deviation
