# Supplementary material for: A survey identifying nutritional needs in a contemporary adult cystic fibrosis cohort
Source: BMC Nutr. 2019 Jan 7;5:4. doi: 10.1186/s40795-018-0266-3 (PMC6474380; doi:10.1186/s40795-018-0266-3)
Supplement: Supplementary file 1 — Survey of nutritional needs. (DOCX 24 kb) [file 40795_2018_266_MOESM1_ESM.docx]

**Additional file 1**

Survey of nutritional needs

The following 14-question survey was designed by our center’s dietician and cystic fibrosis physicians. All patients seen by the RD were asked to complete the survey.

| **1.** | **What are your primary nutrition concerns at this time?**  ***Rank*** *in order of importance.*  *(****1*** ***MOST*** *important,* ***5 LEAST*** *important)* | |  |
| --- | --- | --- | --- |
|  |  | |  |
|  | | ___ Preventing weight loss (or promoting weight gain) | |
|  | | 11 ___ Preventing weight gain (or promoting weight loss) | |
|  | | ___ Nutrition/ food choice education | |
|  | | ___ Blood sugar management  ___ Digestive health/Enzymes | |
|  |  | |  |
| **2.** | **How helpful have visits with the CF dietitian has been over the last two years?**  *(****Circle*** *the best number****. 1 VERY*** *helpful,* ***5 NOT*** *helpful)* | |  |
|  |  | |  |
|  | 1 2 3 4 5 | |  |
|  |  | |  |
| **3.** | **What nutrition programs/offerings would you like to be offered in the CF clinic?**  ***Rank*** *in order of importance.*  *(****1*** ***MOST*** *important,* ***4 LEAST*** *important)* | |  |
|  |  | |  |
|  | ___ CF cookbook based on unprocessed foods | |  |
|  | ___ Information on local food resources (e.g. food banks, meal program) | |  |
|  | ___ Online access to CF Nutrition & Fitness information  ___ Financial assistance for food and oral nutritional supplements like protein drinks and bars | |  |
|  |  | |  |

| **4.** | **Do you have CF Related Diabetes?** |
| --- | --- |
|  | ( ) Yes  ( ) No  ( ) Don’t Know |
| * | **If yes, how do you manage blood sugar levels?**  ***Select ALL*** *that apply* |
|  | ( ) Insulin  ( ) Diet  ( ) Exercise  ( ) None |

| **5.** | **How much time do you, your family, or caregivers spend preparing and cooking food daily?** |
| --- | --- |
|  | ( ) 0-10 minutes  ( ) 11-30 minutes  ( ) 31-45 minutes  ( ) More than 45 minutes |
| **6.** | **Do you supplement your regular diet with oral nutritional supplements?**  *(e.g. protein shakes, smoothies)* |
|  | ( ) Yes  ( ) No  **If yes, what do you use?**  ( ) Smoothies  ( ) Protein bars  ( ) Protein powder  ( ) Scandishake  ( ) Ensure/Boost |
|  |  |
| **7.** | **What do you do for exercise?** |
|  | ( ) Walking for exercise only, not commuting or daily activities  ( ) Running  ( ) Strength Training  ( ) Yoga/Stretching  ( ) Bicycling  ( ) None  **Duration *(on the days you exercise, how many minutes)*** |
|  | ( ) 0-15  ( ) 16-30  ( ) 31-45  ( ) 46-60  ( ) 60+  **Frequency *(days per week)***  ( ) 1  ( ) 2  ( ) 3  ( ) 4  ( ) 5  ( ) 6  ( ) 7 |

| **8.** | **Are you pancreatic sufficient or pancreatic insufficient?**  ( ) Pancreatic sufficient  ( ) Pancreatic insufficient  ( ) Don’t Know  **If pancreatic insufficient, what enzyme brand and strength are you taking?**   \| **Creon** \| 3,000 \| 6,000 \| 12,000 \| 24,000 \| 36,000 \|  \|  \| \| --- \| --- \| --- \| --- \| --- \| --- \| --- \| --- \| \| **Pancreaze** \| 4,2000 \| 10,500 \| 16,800 \| 21,000 \|  \|  \|  \| \| **Zenpep** \| 3,000 \| 5,000 \| 10,000 \| 15,000 \| 20,000 \| 25,000 \| 40,000 \| \| **Ultresa** \| 13,800 \| 20,700 \| 23,000 \|  \|  \|  \|  \| \| **Pertzye** \| 8,000 \| 16,000 \|  \|  \|  \|  \|  \|   How many with meal? _______  How many with snacks? _______  How many total in a day? _______ |
| --- | --- | --- | --- | --- | --- | --- | --- | --- | --- | --- | --- | --- | --- | --- | --- | --- | --- | --- | --- | --- | --- | --- | --- | --- | --- | --- | --- | --- | --- | --- | --- | --- | --- | --- | --- | --- | --- | --- | --- | --- | --- |
|  |  |
| **9.** | **Do you take Vitamins?** |
|  | ( ) Yes  ( ) No    **If yes, what do you use?**  ( ) CF specific vitamin (e.g. AquADEK, MVW Complete, Source CF)  ( ) Over the counter multi vitamin  ( ) Vitamin A  ( ) Vitamin D  ( ) Vitamin E  ( ) Vitamin K |

| **10.** | **Do you take probiotics?**  ( ) Yes  ( ) No  ( ) Don’t Know  **If yes, how often do you take them?**  ***Select BEST*** *option*  ( ) Daily  ( ) Only during exacerbations  ( ) Intermittently  **If yes, which product do you use?** |
| --- | --- |
|  |  |

| **11.** | **How often would you like to see the dietitian?** | | |
| --- | --- | --- | --- |
|  | ( ) 1x year  ( ) 2x year  ( ) Each visit | | |
| **12.** | **Do you ever decline to see the dietitian due to insurance or financial reasons?**  ( ) Yes  ( ) No | | |
| **13.** | **What type of insurance do you have?** | | |
|  | ( ) Medicare/Medicaid  ( ) Commercial (Private) Insurance | | |
|  |  | | |
| **14.** | **What can the dietitian do to help you better meet your nutrition goals?**  *(e.g. creating a meal plan, managing diabetes, weight goals, fitness goals, making your nutrition visit more beneficial)* | | |
|  | |  |  |

**Thank you for taking time to complete this survey.**
